# Impact of previous malignancy at diagnosis on oncological outcomes of upper tract urothelial carcinoma

**DOI:** 10.1186/s12894-023-01206-9

**Published:** 2023-03-29

**Authors:** Hongda Zhao, Kang Liu, Stilianos Giannakopoulos, Emrah Yuruk, Geert De Naeyer, Mario Álvarez-Maestro, Chi-Fai Ng, Pilar Laguna, Jean De La Rosette, Jeremy Yuen-Chun Teoh

**Affiliations:** 1 S.H. Ho Urology Centre, Department of Surgery, The Chinese University of Hong Kong, Hong Kong, China; 2 Department of Urology, Democritus University of Thrace, Alexandroupolis, Greece; 3 Department of Urology, The Ministry of Health, University of Health Sciences, Bagcilar Training and Research Hospital, Istanbul, Turkey; 4 Department of Urology, Onze-Lieve-Vrouw Clinic, Aalst, Belgium; 5 Department of Urology, Hospital Universitario La Paz, Madrid, Spain; 6 Department of Urology, Medipol Mega Hospital, Istanbul Medipol University, Istanbul, Turkey; 7 Department of Surgery, Prince of Wales Hospital, 4/F LCW Clinical Sciences Building, Shatin, New Territories, Hong Kong China

**Keywords:** Previous malignancy, Outcome assessment, Urothelial carcinoma, UTUC, Intravesical recurrence

## Abstract

**Background:**

The evidence of prognostic factors and individualized surveillance strategies for upper tract urothelial carcinoma are still weak.

**Objectives:**

To evaluate whether the history of previous malignancy (HPM) affects the oncological outcomes of upper tract urothelial carcinoma (UTUC).

**Methods:**

The CROES-UTUC registry is an international, observational, multicenter cohort study on patients diagnosed with UTUC. Patient and disease characteristics from 2380 patients with UTUC were collected. The primary outcome of this study was recurrence-free survival. Kaplan-Meier and multivariate Cox regression analyses were performed by stratifying patients according to their HPM.

**Results:**

A total of 996 patients were included in this study. With a median recurrence-free survival time of 7.2 months and a median follow-up time of 9.2 months, 19.5% of patients had disease recurrence. The recurrence-free survival rate in the HPM group was 75.7%, which was significantly lower than non-HPM group (82.7%, *P* = 0.012). Kaplan-Meier analyses also showed that HPM could increase the risk of upper tract recurrence (*P* = 0.048). Furthermore, patients with a history of non-urothelial cancers had a higher risk of intravesical recurrence (*P* = 0.003), and patients with a history of urothelial cancers had a higher risk of upper tract recurrence (*P* = 0.015). Upon multivariate Cox regression analysis, the history of non-urothelial cancer was a risk factor for intravesical recurrence (*P* = 0.004), and the history of urothelial cancer was a risk factor for upper tract recurrence (*P* = 0.006).

**Conclusion:**

Both previous non-urothelial and urothelial malignancy could increase the risk of tumor recurrence. But different cancer types may increase different sites’ risk of tumor recurrence for patients with UTUC. According to present study, more personalized follow-up plans and active treatment strategies should be considered for UTUC patients.

## Introduction

Urothelial carcinomas (UCs) are the fourth most prevalent histologic subtype of cancer in developed countries [1], which can locate in the lower (bladder and urethra) and/or the upper (pyelocaliceal cavities and ureter) urinary tract. However, upper tract urothelial carcinoma (UTUC) is a rare cancer that only accounts for 5–10% of all urothelial malignancies. In western countries, its yearly incidence is around two cases per 100,000 people [2]. Most disease recurrence and progression prevention attempts have focused on improving diagnostic imaging and surgical treatment strategies, with modest efforts on prognostic factors, such as the history of previous malignancy.

Several pre-operative factors have been reported to be associated with the prognosis for patients with UTUC, such as tobacco smoking and gender [2]. However, there is limited evidence to support the role of history of previous malignancy (HPM), especially the history of non-urothelial cancers. According to several publications, a history of bladder cancer before UTUC could significantly increase the rate of intravesical recurrence (IVR) [3, 4]. EAU guidelines on UTUC also set previous bladder cancer as a predictor of increased risk for bladder recurrence [2]. Furthermore, some post-operative factors also have been reported to be associated with oncological outcomes for patients with UTUC, such as intravesical chemotherapy[2].

The Clinical Research Office of the Endourology Society Urothelial Carcinomas of the Upper Tract (CROES-UTUC) registry was established in 2014 [5]. It is one of the most extensive real-world prospective global datasets on the management of UTUC. In this study, we examined the effects of HPM on the risk of tumour recurrence among 996 UTUC patients. We hypothesized that both previous urothelial carcinoma and non-urothelial carcinoma could influence oncological outcomes for patients with UTUC, and different cancer types may increase different sites’ risk of tumor recurrence for patients with UTUC. We believe such information will provide invaluable insights into the impact of HPM on patients with UTUC.

## Methods and material

The CROES-UTUC registry is an international, observational, multicenter cohort study focusing on the management of patients suspected of UTUC [5]. Since the initiation of the registry in November 2014, 101 centres from 29 countries have joined the registry. The registry follows the recommendations of the Agency for Healthcare Research and Quality for the design and use of patient registries for scientific, clinical, and health policy purposes [6]. The study was registered with clinicaltrials.gov (NCT02281188, 02/11/2014) [7], and the study protocol was published before [5].

### Patient selection

Consecutive patients aged ≥ 18 years who had suspected UTUC undergoing any diagnostic or therapeutic surgical intervention were included. Patients with prior history of bladder cancer and other cancer types were not excluded. The study criteria were broad to provide comprehensive real-world data regarding the management and outcomes of patients with suspected UTUC.

### Pathological evaluation

Surgical specimens were processed following standard pathological procedures and reviewed by genitourinary pathologists at each institution. Tumors were staged according to the 2009 American Joint Committee on Cancer/Union Internationale Contre le Cancer TNM classification and graded according to the 2004 World Health Organization classification [8].

### Data Collection and definition

Clinical data on baseline characteristics, risk factors, clinical assessment, intervention received, and survival outcomes were recorded [8]. Data from all participating centres were collected using an online Data Management System. The Data Management System was a web-based system located and maintained at the CROES Office. All urological patients were asked to report their medical history at the time of diagnosis. Patients with a history of urothelial cancers were defined as patients who had urothelial carcinomas before diagnosis. Patients with a history of non-urothelial cancers were defined as patients who had other cancer types before diagnosis.

### Follow-up Regimen

The primary outcome of this study was the overall recurrence-free survival (RFS). And disease recurrence was categorized as any recurrence (local failure in the bladder, upper tract, regional nodes, or distant metastases), bladder-only recurrence (intravesical recurrence), or upper tract-only recurrence. For RFS, participants either had a first recurrence or were censored (no recurrence, deceased with no recurrence or lost to follow-up at the end of the study). Time to recurrence or censoring was calculated by taking the difference between the corresponding date of recurrence (when available) or date of follow-up and the date of the procedure.

### Statistical analysis

Descriptive statistics were calculated for demographic, disease, and smoking characteristics. Associations between categorical variables were assessed using the chi-squared test. Kaplan-Meier (K-M) analysis was performed for recurrence-free survival and the log-rank test determined significance. Multivariate Cox regression analysis was conducted for traditional prognostic factors, including gender, smoking status, pathological stage, and grade. A p-value of < 0.05 was statistically significant. All statistical analyses were performed using SPSS version 26 (IBM Corporation, Armonk, New York) and R studio (RStudio Team, Boston, Massachusetts). Complete case analysis was performed in case of any missing data on the variables of interest.

## Results

### Overview

**Fig. 1:**
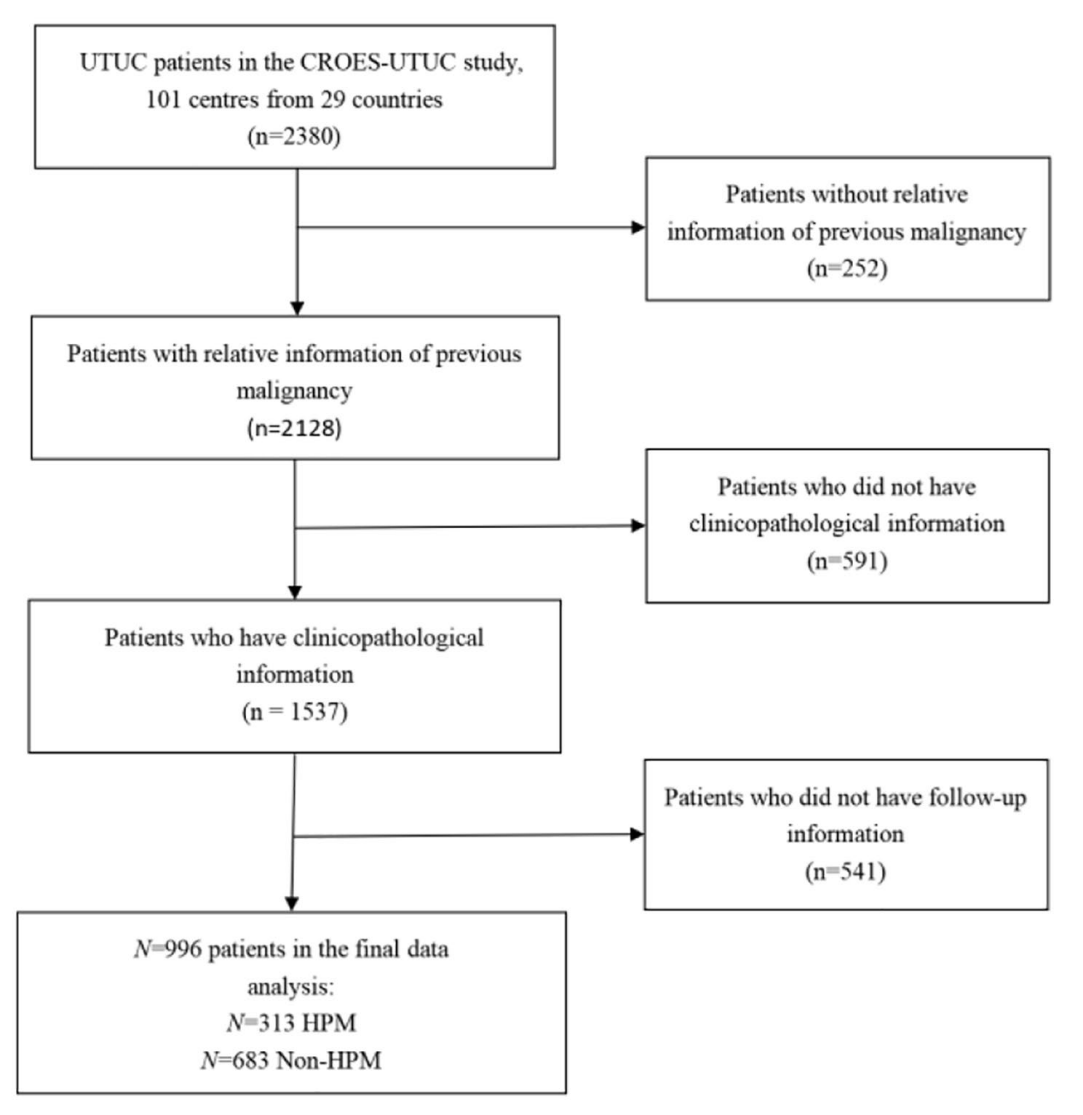
Flowchart describing the selection of UTUC patients who have history of previous malignancy (HPM) versus non-HPM.

A total of 2380 patients were registered in the CROES-UTUC study. 996 patients with UTUC were included in this study, consisting of 313 patients in the HPM group and 683 patients in the non-HPM group (Fig. 1). Table 1 shows the clinicopathological characteristics of the study cohort. Among the 996 UTUC patients, 70.6% of patients were male, with a median (range) age at diagnosis of 73.0 (35.0–94.0) years. About 62.6% of patients were smokers, 6.5% of patients had a family history of positive malignancy, and 2.4% of patients had the risk of occupational hazard. Regarding the pathological tumour stage, 55.9% had pTa/Tis/T1 disease and 44.1% had T2-4 tumor. G1-2 diseases were found in 48.8% and G3 disease was found in 50.4% of patients.

**Table 1:** Baseline characteristics for UTUC patients with or without previous malignant history

	Overall N = 996(%)	HPM N = 313(%)	Non-HPM N = 683(%)	P-value
Age				
Mean(SD)	71.4 (10.78)	72.5 (9.55)	70.8 (11.4)	
Median[Min, Max]	73.0 [35,94.0]	74.0 [35,91.0]	73.0 [39,94.0]	
Missing	260 (26.1)	49 (15.6)	211 (30.9)	
**Sex**				0.211
Male	703 (70.6)	232 (74.1)	471 (69.0)	
Female	292 (29.3)	81 (25.9)	211 (30.9)	
Missing	1 (0.1)	0	1 (0.1)	
**Smoking Status**				0.011*
Smoker	623 (62.6)	216 (69.0)	407 (59.6)	
Nonsmoker	294 (29.5)	73 (23.3)	221 (32.4)	
Missing	79 (7.9)	24 (7.7)	55 (8.1)	
**Family history of positive malignancy**				< 0.001***
Yes	65 (6.5)	27 (8.6)	38 (5.6)	
No	737 (74.0)	199 (63.6)	538 (78.8)	
Missing	194 (19.5)	87 (27.8)	107 (15.7)	
**Occupational hazard**				0.002**
Yes	24 (2.4)	11 (3.5)	13 (1.9)	
No	811 (81.4)	235 (75.1)	576 (84.3)	
Missing	161 (16.2)	67 (21.4)	94 (13.8)	
**pT Stage**				0.003**
Ta/Tis/T1	557 (55.9)	197 (62.9)	360 (52.7)	
T2-4	439 (44.1)	116 (37.1)	323 (47.3)	
**Tumor Grade**				0.702
G1-2	486 (48.8)	158 (50.5)	328 (48.0)	
G3	502 (50.4)	152 (48.6)	350 (51.2)	
GX	8 (0.8)	3 (1.0)	5 (0.8)	
**Procedure**				0.004**
RNU	744 (74.7)	211 (67.4)	533 (78.0)	
URS	198 (19.9)	79 (25.2)	119 (17.4)	
Segmental resection	40 (4.0)	18 (5.8)	22 (3.2)	
Other treatments	14 (1.4)	5 (1.6)	9 (1.3)	

HPM = History of previous malignancy; RNU = Radical nephroureterectomy; URS = Ureterorenoscopy

*p < 0.05; **p < 0.01; and ***p < 0.001

### Association of HPM with clinicopathological characteristics

Among 996 patients, 313 (31.4%) had a history of cancer with urothelial carcinoma as the dominating tumor. Other cancer types include prostate cancer, gastrointestinal cancer, breast cancer, lung cancer, and others (Table 2). Compared with patients without HPM, patients with HPM had a higher rate of family history and occupational hazards (all P < 0.05). Regarding the pathological tumour stage, HPM group had more pTa/Tis/T1 disease and non-HPM group had more T2-4 disease.

**Table 2:** Summary of malignancies diagnosed before UTUC.

	Urothelial carcinoma	Prostate	Breast	Gastrointestinal	Lung	Gyneacological	LYNCH	Multi-typies	Others
Numbers	145	16	13	22	9	11	1	57	36

### Association of HPM with survival outcomes

**Fig. 2:**
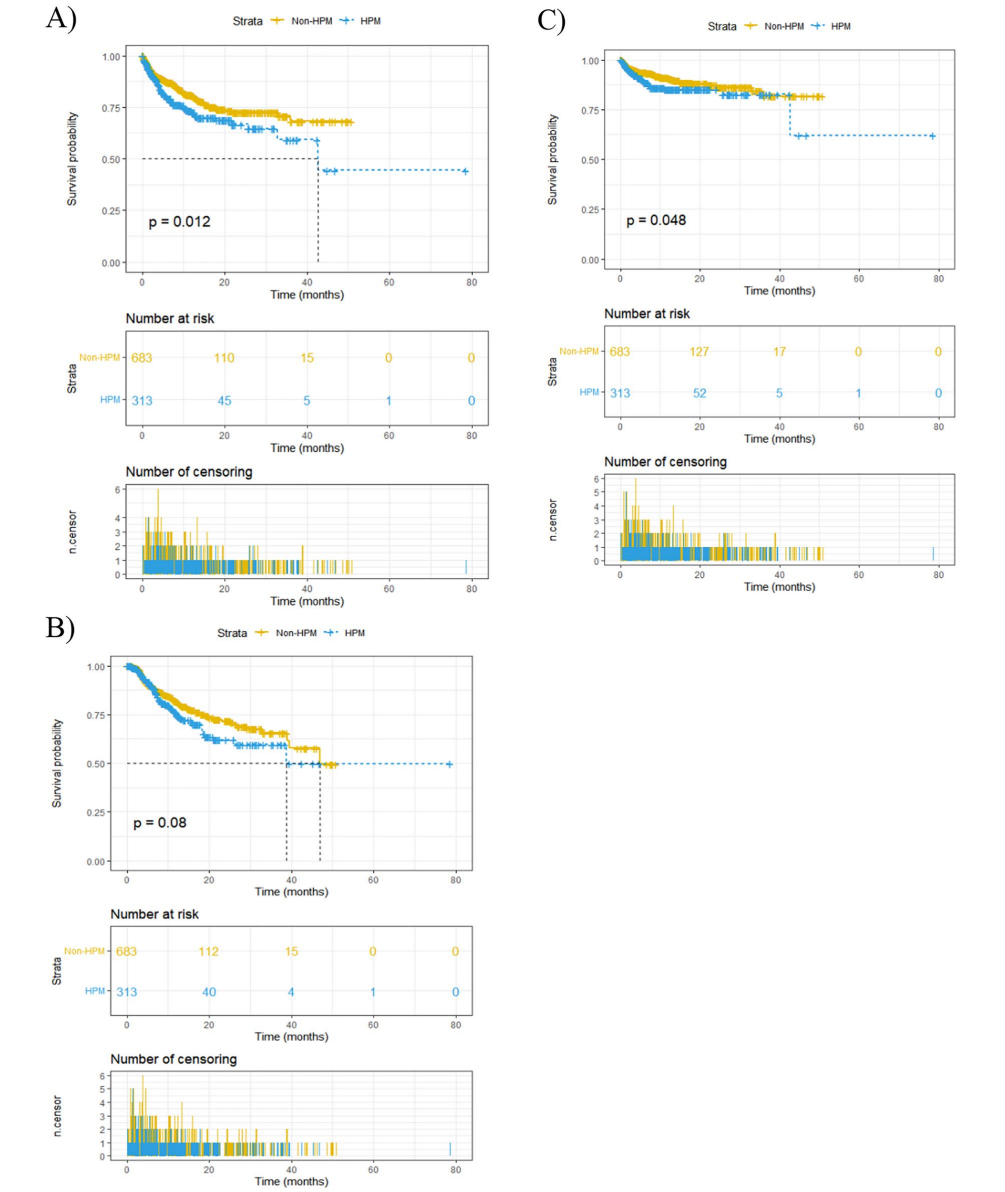
(A) Recurrence-free survival of urothelial carcinoma between UTUC patients with or without previous malignant history. Recurrence-free survival of (B) intravesical recurrence and (C) upper tract recurrence for UTUC patients

The median recurrence-free survival time for patients was 7.2 months and median follow-up time was 9.2 months. And 54 patients ultimately died from UTUC. HPM was shown to be significantly associated with the overall RFS of UCs by K-M analysis (P = 0.012, Fig. 2A). Meanwhile, when only analyzing the intravesical recurrence (IVR) or upper tract recurrence, patients with a history of malignancy had increased chance of developing upper tract recurrence (P = 0.048, Fig. 2C), but not for IVR (P = 0.08, Fig. 2B). In the multivariate cox regression analyses, HPM showed a trend that it was associated with the risk of overall UCs recurrence (P = 0.064, Table 3) after adjusting for other prognostic factors. Besides that, advanced pT stage was also associated with decreased RFS (P = 0.011, Table 3). When separately analyzing IVR and upper tract recurrence, the results also showed a trend that HPM might be associated with upper tract recurrence (P = 0.067, Table 3).

**Table 3:** Multivariate Cox regression analyses on recurrence-free survival of urothelial carcinoma, intravesical recurrence and upper tract recurrence in patients with or without previous malignant history

	Urothelial carcinoma		Intravesical recurrence		Upper tract recurrence	
	HR(95% CI)	P value	HR(95% CI)	P value	HR(95% CI)	P value
**Smoke**		0.052		0.585		0.264
No	Reference		Reference		Reference	
Yes	1.426 (0.996–2.041)		0.907 (0.639–1.287)		1.333 (0.805–2.205)	
**Gender**		0.110		0.738		0.131
Female	Reference		Reference		Reference	
Male	0.759 (0.541–1.064)		1.064 (0.739–1.531)		0.698 (0.437–1.113)	
**Previous malignancy**		0.064		0.114		0.067
No	Reference		Reference		Reference	
Yes	1.336 (0.984–1.814)		1.300 (0.939–1.799)		1.486 (0.972–2.273)	
**pT Stage**		0.011*		0.685		0.111
Ta/Tis/T1	Reference		Reference		Reference	
T2-4	1.605 (1.116–2.309)		1.079 (0.746–1.562)		0.648 (0.381–1.104)	
**Tumor Grade**		0.436		0.401		0.019*
G1-2	Reference		Reference		Reference	
G3	0.872 (0.617–1.231)		0.855 (0.594–1.232)		1.842 (1.103–3.075)	

CI = confidence interval; HR = hazard ratio;

*p < 0.05; **p < 0.01; and ***p < 0.001

**Fig. 3:**
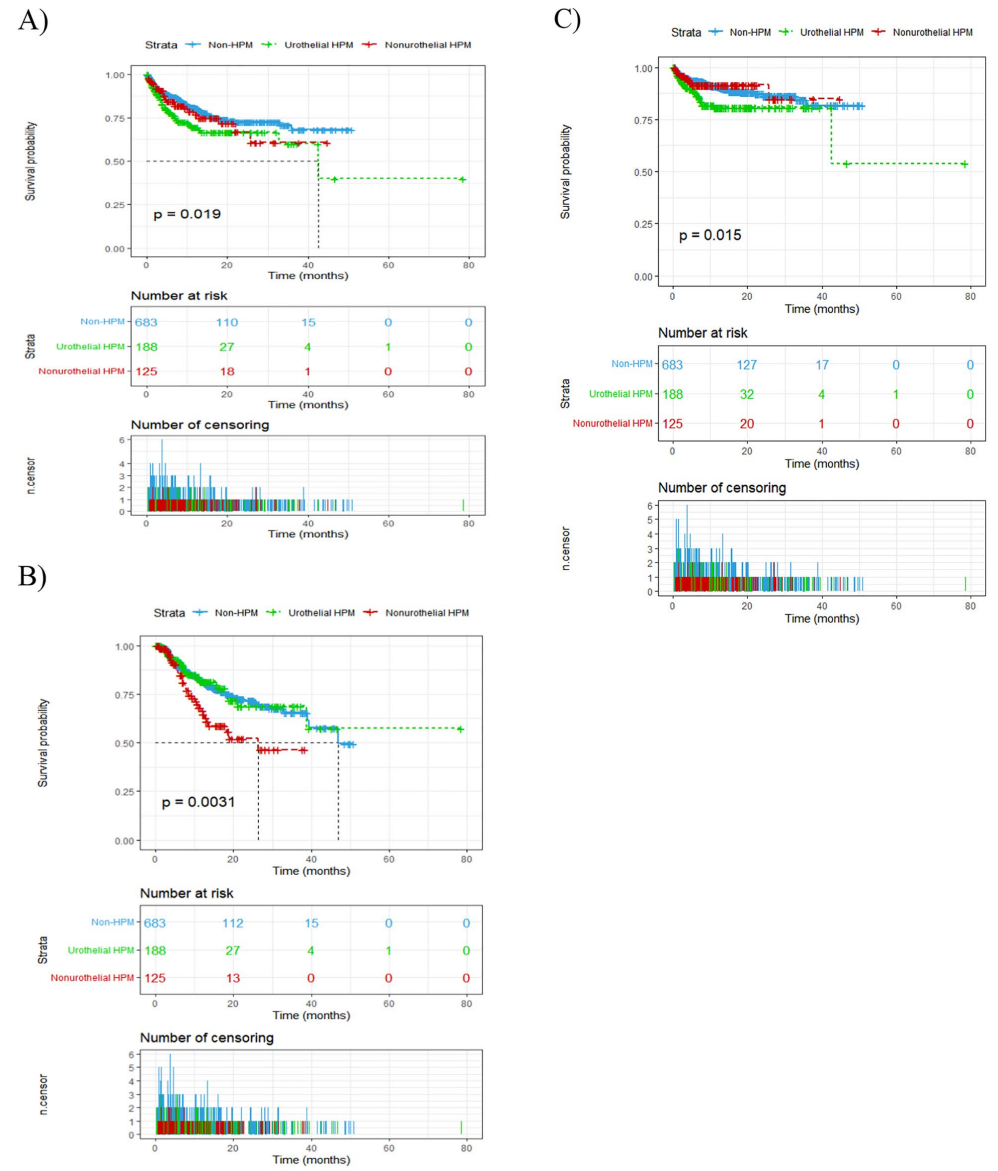
(A) Recurrence-free survival of urothelial carcinoma in patients between three groups, history of urothelial cancers (UTUC or bladder) vs. history of non-urothelial cancers vs. no history of any cancer. Recurrence-free survival of (B) intravesical recurrence and (C) upper tract recurrence

We further analysed the effect of different types of previous malignancy on cancer recurrence, by dividing patients into 3 groups, with a history of UCs, with a history of non-urothelial cancers, and those with no history of any cancer. We found that both previous UCs and non-urothelial cancers could increase the risk of UCs recurrence (P = 0.019, Fig. 3A). In addition, patients with a history of non-urothelial cancer had the highest risk of IVR (P = 0.003, Fig. 3B), and patients with a history of UCs had the highest risk of upper tract recurrence (P = 0.015, Fig. 3C). After adjusting for traditional prognostic factors in a multivariate cox regression analysis, previous non-urothelial cancers remained independent predictors of IVR (P = 0.004, Table 4), and previous urothelial cancers remained independent predictors of upper tract recurrence (P = 0.006, Table 4).

**Table 4:** Multivariate Cox regression analyses on recurrence-free survival of urothelial carcinoma, intravesical recurrence and upper tract recurrence in patients between three groups, history of urothelial cancers (UTUC or bladder) vs. history of non-urothelial cancers vs. no history of any cancer

	Urothelial carcinoma		Intravesical recurrence		Upper tract recurrence	
	HR(95% CI)	P value	HR(95% CI)	P value	HR(95% CI)	P value
**Smoke**		0.058		0.695		0.312
No	Reference		Reference		Reference	
Yes	1.416 (0.988–2.029)		0.993 (0.657–1.323)		1.298 (0.783–2.154)	
**Gender**		0.102		0.540		0.084
Female	Reference		Reference		Reference	
Male	0.754 (0.537–1.058)		1.121 (0.778–1.616)		0.660 (0.412–1.057)	
**Previous malignancy**		0.151		0.013*		0.013*
Non-HPM	Reference		Reference		Reference	
Uro-HPM	1.408 (0.985–2.014)	0.061	0.981 (0.641–1.502)	0.931	1.938 (1.214–3.095)	0.006**
Non-uro-HPM	1.229 (0.794–1.901)	0.355	1.810 (1.206–2.719)	0.004**	0.874 (0.430–1.777)	0.710
**pT Stage**		0.012*		0.760		0.139
Ta/Tis/T1	Reference		Reference		Reference	
T2-4	1.598 (1.111-2.300)		1.059 (0.732–1.534)		0.668 (0.391–1.140)	
**Tumor Grade**		0.419		0.537		0.013*
G1-2	Reference		Reference		Reference	
G3	0.867 (0.613–1.226)		0.891 (0.617–1.286)		1.916 (1.145–3.204)	

CI = confidence interval; HR = hazard ratio; PM = History of previous malignancy

*p < 0.05; **p < 0.01; and ***p < 0.001

## Discussion

As one of the most extensive studies investigating the impact of HPM on oncological outcomes of patients with UTUC, our results showed that previous malignancy was significantly associated with an increased risk of tumour recurrence. Among the 996 patients with UTUC, 313 patients reported a history of prior malignancy. The Kaplan-Meier analysis showed that both previous urothelial and non-urothelial cancers could negatively affect the RFS of patients with UTUC. Unlike previous studies[3, 9], our results showed that patients with a history of UCs would have a higher risk of upper tract recurrence, and patients with a history of non-urothelial cancers would have a higher risk of IVR. After adjusting for traditional prognostic factors in a multivariate cox regression analysis, uro-HPM remained a significant association with upper tract recurrence and non-uro-HPM remained a significant association with IVR. This means different types of HPM could cause different results. And the multivariate analyses of the whole HPM also showed a trend that HPM weighed an impact upon oncological outcomes.

For the impact of HPM, most studies focus on the previous bladder cancer of UTUC patients. According to the EAU guideline, patients with previous radical cystectomy for high-grade bladder cancer were stratified as the high-risk group [2]. A meta-analysis that identified significant predictors of bladder recurrence after RNU also suggested previous bladder cancer as a predictor of increased risk for IVR [3]. Shuxiong et al. also reported that previous or simultaneous bladder cancer was a significant predictor of worse prognosis [4]. However, our results showed that patients with previous urothelial cancers would have a higher risk of upper tract recurrence. Several factors were causing this heterogeneous result. First of all, for patients with previous UCs, around 20% of them experienced intravesical chemotherapy after RNU. And this rate is significantly higher than the rate for patients with non-urothelial cancers, thus confirming that intravesical chemotherapy could reduce the rate of IVR. In addition, our analysis firstly included patients with previous UTUC, thus enhancing the risk of local recurrence. Based on our results and earlier studies, the history of previous urothelial carcinoma could not only enhance the risk of IVR, but also increase the risk of upper tract recurrence.

In addition, we firstly analyzed the impact of previous non-urothelial cancers on oncological outcomes. According to our results, we found that previous non-urothelial cancers could significantly increase the risk of UCs recurrence, especially the IVR. Ryohei et al. also reported that non-urothelial malignant history could increase the risk of recurrence for patients with non-muscle invasive bladder cancer (NMIBC) [10]. We think the main reason for this situation is the field change cancerization effect [11]. As we know, some environmental risk factors and living habits could increase the risk of tumour occurrence and recurrence for non-urothelial cancers, such as smoking and chemical exposure. These carcinogens are metabolized and excreted through the urine [12]. As the organ stores urine, the urinary bladder is maximally exposed to carcinogens compared with the renal pelvis and ureter [13]. When the carcinogens are excreted into the urine and stored in the bladder, they could induce genetic mutation in an exposed field, thus enhancing the risk of tumour occurrence and recurrence [14]. In Ryohei’s study, they also found that smokers would have a higher risk of being influenced by previous non-urothelial malignant history [10].

In addition, recent genetic research revealed that genetic instabilities could cause many forms of cancer to grow concurrently or metachronously, as well as accelerate tumor recurrence in some cancers with a history of malignant tumors. The mutation of CDKN2A was reported to elevate the risk of multiple cancers [15]. And the homozygous deletions in CDKN2A were also associated with the oncological outcomes for NMIBC patients [16]. Besides that, a study reported that prostate cancer was an unfavorable factor for intravesical recurrence-free survival [17]. Lombard et al. also found that androgen receptors were highly expressed in the bladder and could promote bladder cancer progression [18].

There are several inherent limitations to this study. First, as a non-randomized study,

there exists some inherent bias when evaluating the treatment procedure. Second, as a multi-center study, it is difficult to standard the clinical practices and operating decisions. This introduces heterogeneity, and some results may be challenging to interpret. Third, the follow-up surveillance protocol was not standardized and was subject to the discretion of the participating centre. This might affect the accuracy and precision of the follow-up survival data. And as a non-randomized prospective registry, there exists some problems of data collection, thus causing many missing data which may affect the accuracy of results. The limitations that many patients were lost to follow-up at the end of the study might influence the level of evidence. In addition, the short follow-up time might also influence the accuracy of this study, but we think that our study described some differences statistically and hope these results can generate hypotheses for future studies. In summary, although the registry is not devoid of limitations, its strength mainly relies on its design, based on a prospective registry conducted with a common protocol.

## Conclusion

Based on the CROES-UTUC registry, our results support the hypothesis that HPM could increase the risk of recurrecnce for patients with UTUC. Furthermore, the study first identified the effect of previous non-urothelial malignancy on bladder recurrence and the impact of previous urothelial malignancy on the upper tract recurrence. This new finding suggests that urologists should consider more personalized follow-up plans and take more active treatment strategies, such as post-operation intravesical chemotherapy and regular cystoscopy.

## Data Availability

The datasets used and/or analysed during the current study available from the corresponding author on reasonable request.
